# One-pot simultaneous production and sustainable purification of fibrinolytic protease from *Bacillus cereus* using natural deep eutectic solvents

**DOI:** 10.1038/s41598-020-70414-2

**Published:** 2020-08-07

**Authors:** Senthil Kumar Rathnasamy, Aadhavan Durai, A. A. Vigneshkumar, C. Purushothaman, Devi Sri Rajendran, K. Chandramouliswaran

**Affiliations:** Green Separation Engineering Laboratory, School of Chemical and Biotechnology, SASTRA Deemed to be University, Thanjavur, Tamil Nadu 613401 India

**Keywords:** Biotechnology, Engineering

## Abstract

The present study report for the first time on the one-pot production and purification of fibrinolytic protease from *Bacillus cereus* by extractive fermentation using natural deep eutectic solvents (NADES). Cheese whey was chosen as a sustainable low-cost production alternative yielding a significantly high amount of protease (185.7 U/mg). Five natural deep eutectic solvents with menthol as hydrogen bond donor and sugar molecules as corresponding hydrogen bond acceptors were synthesized and their association was confirmed with H^1^ NMR. Thermophysical investigation of the synthetic NADES was accomplished as a function of temperature to define their extraction ability. Response surface methodology based optimization of concentration of NADES (77.5% w/w), Na_2_SO_4_ (14% w/v) and cheese whey (1% w/w) were accomplished for extractive fermentation. Further, preparative purification using size exclusion chromatography was used to quantify the amount of enzyme obtained in the extraction phase (190 U/ml). On subsequent purification with an anion exchange column, the maximum purity fold (21.2) with enzyme activity (2,607.8 U/ml) was attained. The optimal pH (8.0), temperature (50 °C) were determined and the in-vitro fibrinolytic activity has been confirmed using a fibrin plate assay.

## Introduction

Modernization of lifestyle has led to increased incidence of cardiovascular diseases making them life-threatening. A recent study shows that about 31% of global mortality is due to this cardiovascular diseases^1^. The major cause of the cardiovascular disease is found to be the block occurring in blood vessels due to the incomplete lysis of blood clots. This process initiates with the conversion of the precursor fibrinogen by enzyme thrombin to its activated form fibrin which crosslinks to form blood clot^2^. As soon as new tissue is formed over the wound, the blood clot should be hydrolyzed into smaller fibrin threads by the enzyme plasmin. Plasmin is formed from plasminogen by the activity of tissue plasminogen activator (t-PA)^3^. Malfunction of these enzymes is the key factor involved in the incomplete lysis of fibrin clots. These complexities could be overcome by treatment with commercial anticoagulants like streptokinase and urokinase. Frequent dosage of this enzyme causes thrombophilia^4^ and internal hemorrhage on the walls of the intestine^5^. Usage of natural counterparts like-nattokinase possesses various complications including low fibrin specificity. Regular consumption of nattokinase results in elevated blood plasmin that decrease the availability of blood coagulation factors^6^. Recently, more research focused on the production and purification of fibrin specific anticoagulants from various microbial sources such as bacteria, fungi and algae species. *Bacillus cereus*, a well-studied microbial isolate reported as a potential source for the production of target-specific fibrin digesting enzyme and have been investigated as the model organism for decades^7,8^. Eventhough these organisms produce high specific fibrinolytic protease, the yield and purity of these enzymes are greatly influenced by the purification techniques employed to isolate them^9,10^.

Industrial production of these therapeutically important microbial enzymes remain unrealized due to two major factors remaining unaddressed: one being the higher cost of production media which seeks utilization of alternative low-cost media rich in essential primary metabolites. Usage of low-cost media guarantees to meet the requirement of large scale industrial consumption and provides improved yield with the extensive choice of materials like whey^11^, corn steep liquor^12^ and oil cakes^13^. Additionally, the requirement of multistep purification protocols for attaining ultrapure nature of product adds up a major component of cost. Conventional purification steps include techniques like ammonium sulphate precipitation and ultrafiltration. These are less specific and their harsh environment influences the nativity of protein and enforces them to lose their activity. Further enhancement in purity is accomplished with various chromatographic techniques such as anion exchange chromatography and gel filtration chromatography^2^. Employing these chromatography based purification techniques results in the elevation of the product cost. The extractive fermentation process provides an alternative approach for these conventional purification techniques by integrating both extraction and fermentation as a single unit operation. The extractant is supplied post log phase of production to separate the active enzyme produced. This leads to the approach of simultaneous production and purification into the limelight by employing an aqueous biphasic system (ABS) for product recovery. This aqueous biphasic method facilitating the deposition of cell debris in the bottom phase which also eliminates the use of high-speed centrifugation^14^. Conventional process in the formation of ABS systems involves the addition of two polymers (or) a polymer and salt at a certain concentration which forms immiscible two-phase. Solutes of variable sizes such as ions, chemical moieties, biomolecules, and even cells can be separated in this system on their immiscible phases^15^.

Recently ionic liquids are being used as effective substituents over polymers in two-phase extraction due to their selectivity. Ionic liquids are capable of acting as extracting agents due to their salting-out property with various metallic salts in their ionic state. However, its application becomes trivial due to various disadvantages like the high cost of synthesis, toxic in nature and variable recovery. Deep eutectic solvents (DES) are the modern age solvents used for the extraction of commercially important therapeutic enzymes^16,17^, occurs as the homogenous mixture of hydrogen bond acceptors (HBA) and hydrogen bond donors (HBD)^18,19^. Various types of DES have been established based on the choice of HBA and HBD of which ampiphilic^20^, protic^21^ and natural^16^ deep eutectic solvents are commonly known types. Natural derivatives like carbohydrates^22^, naturally occurring secondary metabolites like betaine^23^, glycerol^24^ and biologically obtained acids like citric acid^25^ are used as HBA and HBD to form natural deep eutectic solvents (NADES)^17^. Using these natural deep eutectic solvents for extraction of target molecule maintains nativity of the product and improves the recycling capability of the corresponding participant molecules. This new mechanism of using NADES with Na_2_SO_4_ in aqueous two-phase helps in the recovery of these therapeutic high-value products in their native state. NADES has been studied for the processing of biomass and improved saccharification low toxicity, easy recycling and biodegradability^26^. Extraction of various commercial and therapeutic compounds by ABS with NADES as primary solvent andNa_2_SO_4_ for forming immiscible two-phase have been established for protease^16^ and immunoglobulins^17^. NADES have been proven to be biocompatible extraction medium maintaining the activity, stability and structure of laccase^23^.

**Figure 1:**
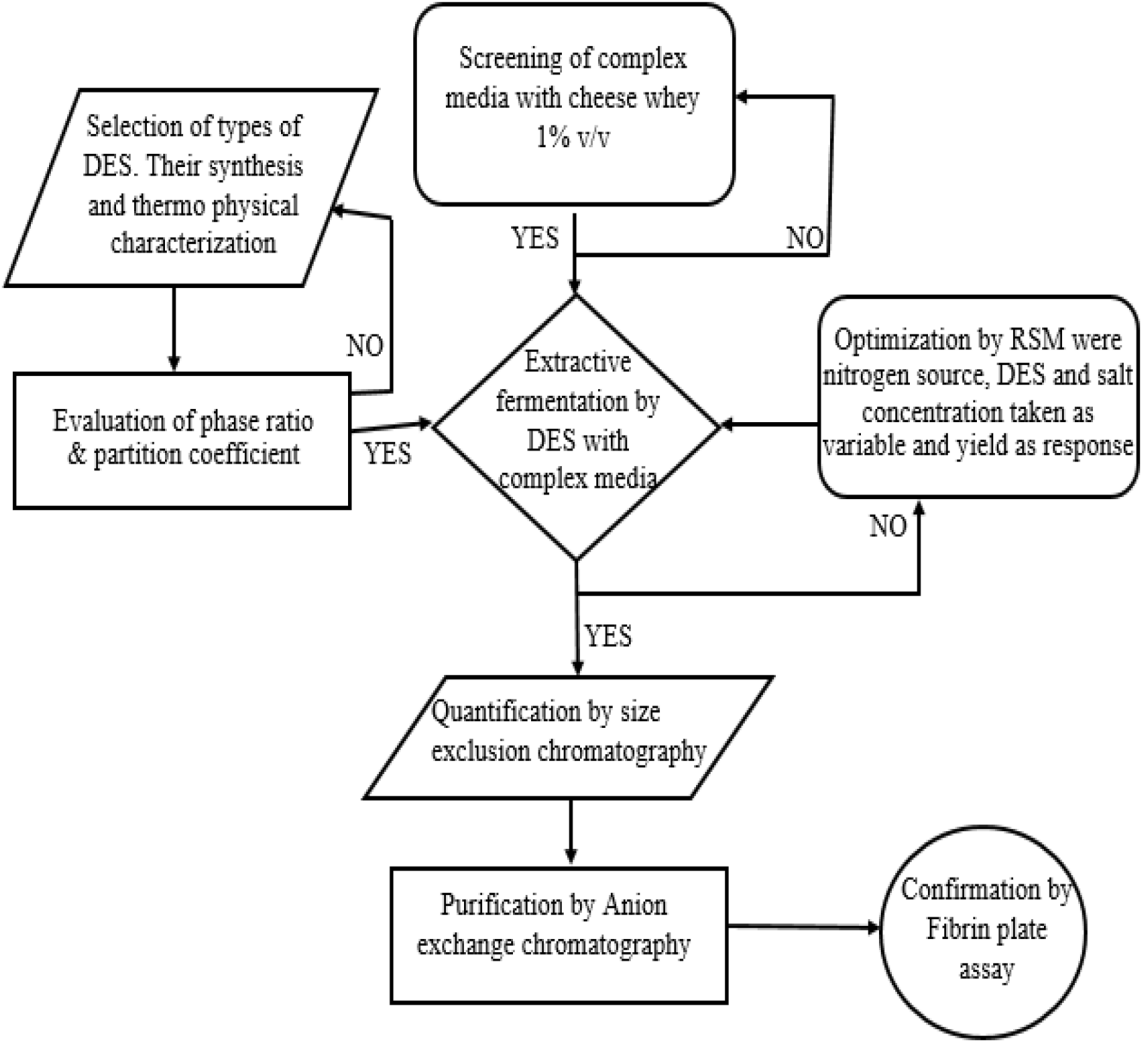
Schematic representation of an overall process of extractive fermentation, production and purification of fibrinolytic protease.

This study aims at the one-pot simultaneous production and purification of therapeutically significant fibrinolytic protease by the integration of fermentation with aqueous biphasic systems formed using NADES and salt. Four low-cost alternatives (cheese whey, cane molasses, steamed rice water and rotten egg) were screened for maximum production of protease. Six NADES were synthesized and their thermophysical properties were studied in variable temperature conditions. The binodal curve of these NADES with Na_2_SO_4_ as the primary salting-out agent was determined to evaluate their extraction efficiency. The partition coefficient of these NADES was observed by performing extractive fermentation of fibrinolytic protease with Na_2_SO_4_. The extracted protein was quantified using size exclusion chromatographic column under optimized conditions. The purity of the fraction collected will be enhanced by anion-exchange chromatography. The ultrapure enzyme aliquot was screened for fibrinolytic activity by observing a zone of clearance in fibrin plate. The optimal activity conditions (pH and Temperature) of the purified enzyme was evaluated. The overall schematic representation of this work is explained in Fig. 1.

## Result and discussion

### Screening of complex medium

*B. cereus* produce a variable amount of protease based on the type and composition of production media used. Cheese whey supported the maximum production of an active proteolytic enzyme (185 U/mg) compared to molasses (149 U/ml), rice water (108 U/ml) and rotten egg (146 U/mg) (Table 1). Cheese whey, a by-product from cheese industries is rich in essential nutrients and minerals and is available in plenty thus could be exploited for the production of valuable industrial products. Generally, fast metabolizing carbon source does not support enhanced protein production (Bijender). Among various carbon sources, rice water yields low protease production from *B. cereus* due to the presence of simply digestible polysaccharide (starch) that promotes cell density rather than secondary metabolite production. The rotten egg being one of the most promising nitrogen sources showed lesser protease yield because fast metabolizable protein sources stop releasing nitrogen before attaining log phase. Apart from being a rich source of nitrogen, cheese whey is inherent in trace elements such as Mg, Ca, Zn, S, Cu, Mn which promotes moderate metabolism and high secondary metabolite production. Further, cheese whey exhibits slow release of nitrogen which intensifies protease production.

**Table 1:** Various substrate used as source of the complex media and the activity of the enzyme produced during fermentation is calculated as shown.

Substrate	Enzyme activity (U/ml)
Molasses	149 ± 1.8
Rice water	108 ± 1.2
Cheese whey	185.7 ± 3.4
Rotten egg	146 ± 2.3

### Thermophysical evaluation of NADES

#### Density

**Figure 2:**
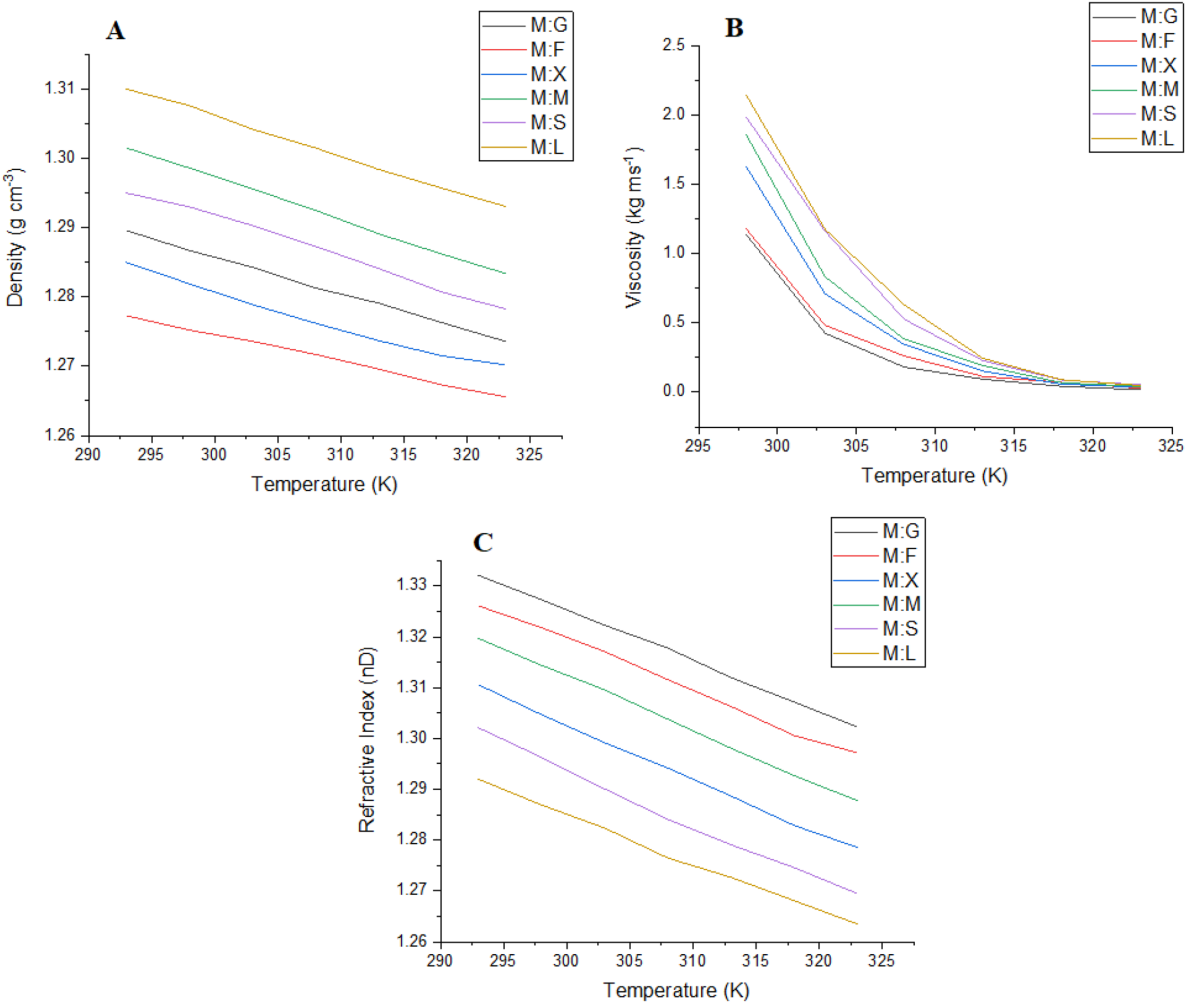
Temperature dependence (273–373 K) variation of the thermophysical parameters like density (**A**), viscosity (**B**) and refractive index (**C**) for all NADES [M:G (menthol:glucose), M:F (menthol:fructose), M:X (menthol:xylose), M:M (menthol:maltose), M:S (menthol: sucrose), M:L (menthol:lactose)].

The density of the NADES has a significant impact on the extraction of the target molecules. NADESs are subjected to density measurement as the function of temperature. A linear dependence of temperature is observed to occur with the reduction in density of all the NADESs (Fig. 2A). NADES with the lowest density is highly susceptible to large salt consumption during phase formation thus have reduced yield of target molecules. A higher density is relative to high saturation and reduced space for target protein interaction hence exhibits low affinity to the product. The destabilization of the hydrogen bonds with an increase in the temperature leads to an increase in entropy^24^ thus reducing the density of corresponding NADES. The thermophysical characteristics of these NADESs and their relativity on extraction coefficient were reported earlier^27^. The order of decreasing density remains the same as M:X > M:F > M:G > M:S > M:M > M:L at both higher and lower temperature range. The values of density and temperature are related by the following equation.

1 $$ \rho = X_{1} T + X_{2} $$

where denotes temperature in Kelvin and density denoted by ρ (kg/m^3^) while X_1_ and X_2_ denote the linearity coefficients whose values are provided in Table 2.

**Table 2:** Temperature relative parameter evaluated for density, viscosity, conductivity and refractive index (n = 3, mean values represented along with standard deviation) X_1_ and X_2_ denote linearity coefficients of density, R is the gas constant, μ_0_ and K^0^ are viscosity and conductivity of liquid in vacuum and Eμ and E_K_ are activity coefficients corresponding to viscosity and conductivity respectively.

NADES	Density (g/cm^3^)		Viscosity (mPa s)		Refractive index (n_D_)	
	X_1_	X_2_·10^−4^	μ_0_	E μ/R	s	u
M:G	1.345	− 6	3.65 × 10^−7^	− 5,814.28	143.63	− 0.345
M:F	1.310	− 6.32	3.8 × 10^−13^	10,582	106.9	− 0.31
M:X	1.317	− 6.40	7.85 × 10^−20^	− 15,335	123.67	− 0.297
M:M	1.356	− 5.58	1.38 × 10^−3^	− 9,855	133.67	− 0.34
M:S	1.369	− 7.44	2.8 × 10^−9^	− 7,645.3	138.71	− 0.345
M:L	1.375	− 7.56	3.8 × 10^−8^	− 8,541	146.33	− 0.341

#### Refractive index

The refractive index of all the NADES is measured as the function of temperature. Similar to density, the refractive index of all NADES under investigation shows linear dependence with temperature. An increase in mobility leads to a decrease in the refractive index of the NADES. The reduction of the interaction between the molecules tends to increase the free volume thus lowering the refractive index at higher temperatures. In one of our earlier investigations, FDESs have been observed to exhibit similar delineation with varying temperature^28^. The higher refractive index is exhibited by NADES formed with higher molecular weight counterparts. This is due to sustained non-covalent interaction between the molecules even at a higher temperature. Therefore, it could be concluded that denser molecules have higher intermolecular interactions thus are low in extraction ability (Fig. 2C). The order of refractive index for NADES under investigation is as follows: M:S > M:M > M:L > M:F > M:G > M:X. The observed trends of refractive index are associated with the temperature by the following equation.

2 $$ n_{D} = V_{1} T + V_{2} $$

where V_1_ and V_2_ indicate the linearity coefficients whose values are provided in Table 2 while T is the temperature in Kelvin and the refractive index is denoted by n_**D**_^28^.

#### Viscosity

The viscosity of all NADESs is measured as a function of the temperature and reveals its dependence on the choice of HBD. Non-Linear dependence of viscosity over the increasing temperature is exhibited by NADES as shown in (Fig. 2B). The highest value recorded is 1,094 kg/m s and the lowest value was recorded as 125 kg/m s. The order of viscosity is dependent on the side chain length of corresponding HBD and is represented as follows (M:S > M:X > M:M > M:F > M:G > M:L). This effect is similar to our earlier reports describing the liberalization of an associated long-chain moiety of HBD and HBA at higher temperature thus proportionating decrease in non-covalent interactions^29^.

3 $$ \eta = \eta_{0} e^{{ - \frac{{E_{\eta } }}{RT}}} $$

where η is the viscosity, η_0_ and E_η_ indicate variable parameters whose values are provided in Table 2^30^.

#### Binodal curve determination

**Figure 3:**
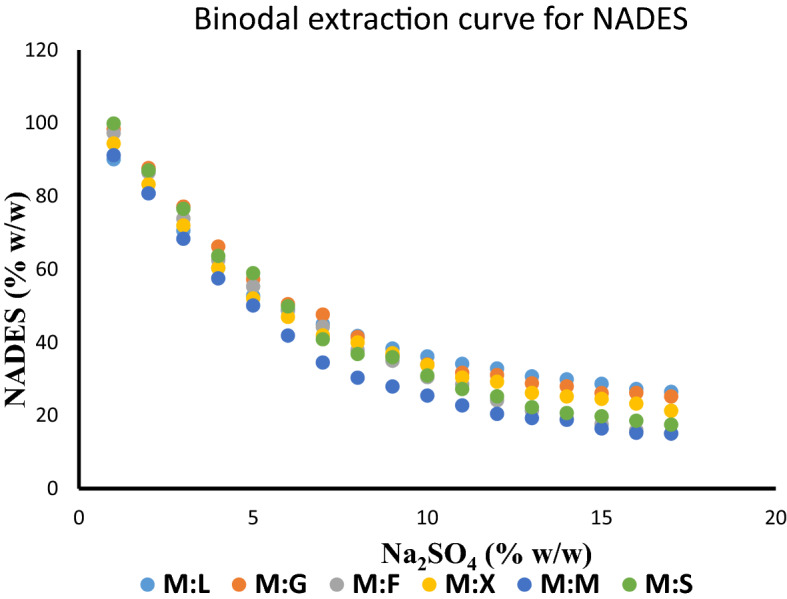
Binodal curve of various NADES in combination with Na_2_SO_4_ to determine its corresponding extraction coefficient.

Binodal curves for all NADES was constructed by cloud point method. The determination of the binodal curve provides a clear interpretation of the extraction ability of individual NADES. The plot showed that NADES with higher density has extended binodal curve due to their extensive salting-out capability. In contrast, NADES with low density tends to lose their phase volume with less amount of salt and hence represent a shallow two-phase region. Those eutectic solvents with moderate density are observed to carry a decent biphasic region thus exhibit large salting-out capability (Fig. 3). This could be the result of the difference in facilitating the entropy change thereby influencing the stability of the ATPS system in combination of each NADES with corresponding salt. The order of phase formation is observed to occur similar to the order of phase ratio and represented as follows M:L < M:G < M:F < M:X < M:S < M:M.

#### H^1^ NMR analysis of NADES

The H^1^ NMR analysis of NADES provides insights into the association pattern and the nativity of both HBA and HBD involved in the investigation. The graphs (Figs. S1–S6) provides the occurrence of sharp peaks denoting the bonding pattern of various molecules associated. It could be observed that peaks responsible for the ionic structure of both HBA and HBD occurs along with other indistinct peaks which could be the resultant of the non-covalent interaction between these molecules. Further, displacement of the peak was found to occur due to the stability in the non-covalent interactions occurring at the eutectic temperature.

### Effect of salt choice and NADES

**Figure 4:**
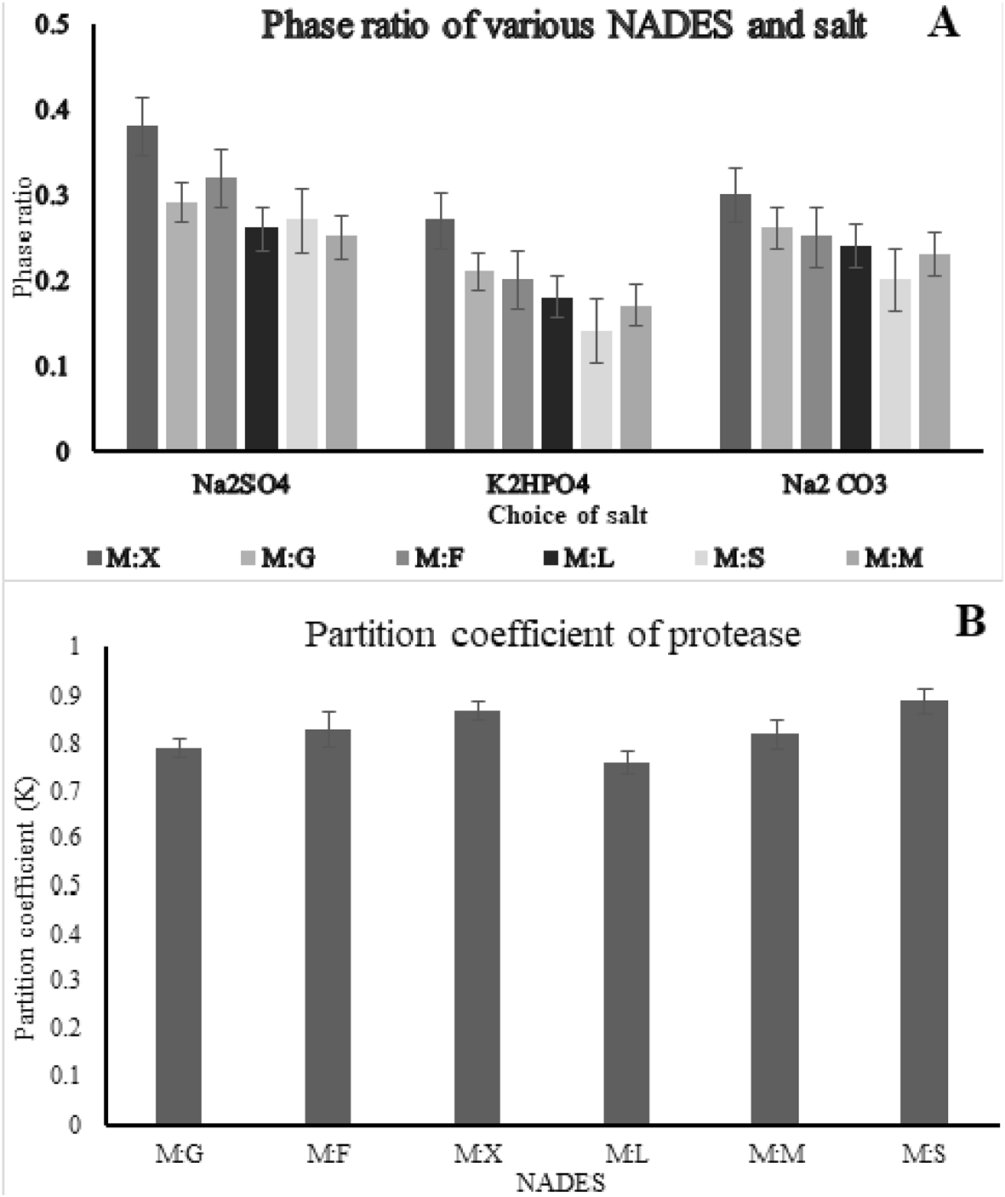
The influence of the choice of NADES and salt concentration on the yield of the fibrinolytic enzyme is denoted by both phase ratio (**A**) and partition coefficient (**B**). It could be observed that the yield differs based on the choice of NADES used which signifies they are selective towards the extraction.

Selection of suitable halting out agent affects the capacity of NADES to form aqueous two-phase and influences its corresponding phase volume ratio. The role of salts in the formation of phases depends on their ionization capability. Salt with high ionization constant rapidly forms phase than the counterparts with a lower degree of ionization. Increase in the size of the ionic moiety lowers the probability of its interference with other phase forming solvents. Therefore the volume and concentration of salt required for the formation of cloud point are very low for large ionic size counterparts. The order of the phase formation of salt is Na_2_CO_3_ > Na_2_SO_4_ > K_2_HPO_4_ (Fig. 4A). Na_2_SO_4_ exhibits a reasonable phase volume and provides high selectivity towards the isolation of the target molecule. This is because Na_2_SO_4_ has moderately heavy cation with high ionization capacity rendering energy required for facile phase formation.

The specificity NADES towards isolation of the target molecule is determined using the pre-inoculated broth rich in protease. This task-specific capability of NADES could be related to its thermophysical ability. NADES with moderate density and viscosity have larger phase ratio providing sufficient room for extraction of the target molecule. On the contrary, low-density NADES needs a large amount of salt hence exhibit low extraction coefficient. Similar interference could be observed with the usage of denser NADES due to the low availability of interstitial space accommodating limited amount of target molecule. This inherently reduces the recovery of product to the top phase. (Fig. 4B) shows the order of partition coefficient for the NADES (M:L < M:G < M:M < M:F < M:X < M:S) exhibiting similarity with the ascending order of their density. NADES (M:S) with moderate density shows the highest partition coefficient (0.89) in combination with Na_2_SO_4_.

Addition of NADES and Na_2_SO_4_ to the pre-incubated broth would engage phase formation within the available product. Further, this is the first investigation to best of our knowledge that used NADES in a crude broth to isolate fibrinolytic protease. The effective phase partitioning of fibrinolytic protease alone has occurred besides the presence of various other undesirables (cell debris and other proteins) in the broth (devoid of any clarification steps). Further, usage of NADES for aqueous two-phase formation which is a recyclable and naturally derived solvent that is devoid of any traces for toxicity ensures a sustainable way of recovering the product.

### Optimization of process variables in extractive fermentation using NADES

**Figure 5:**
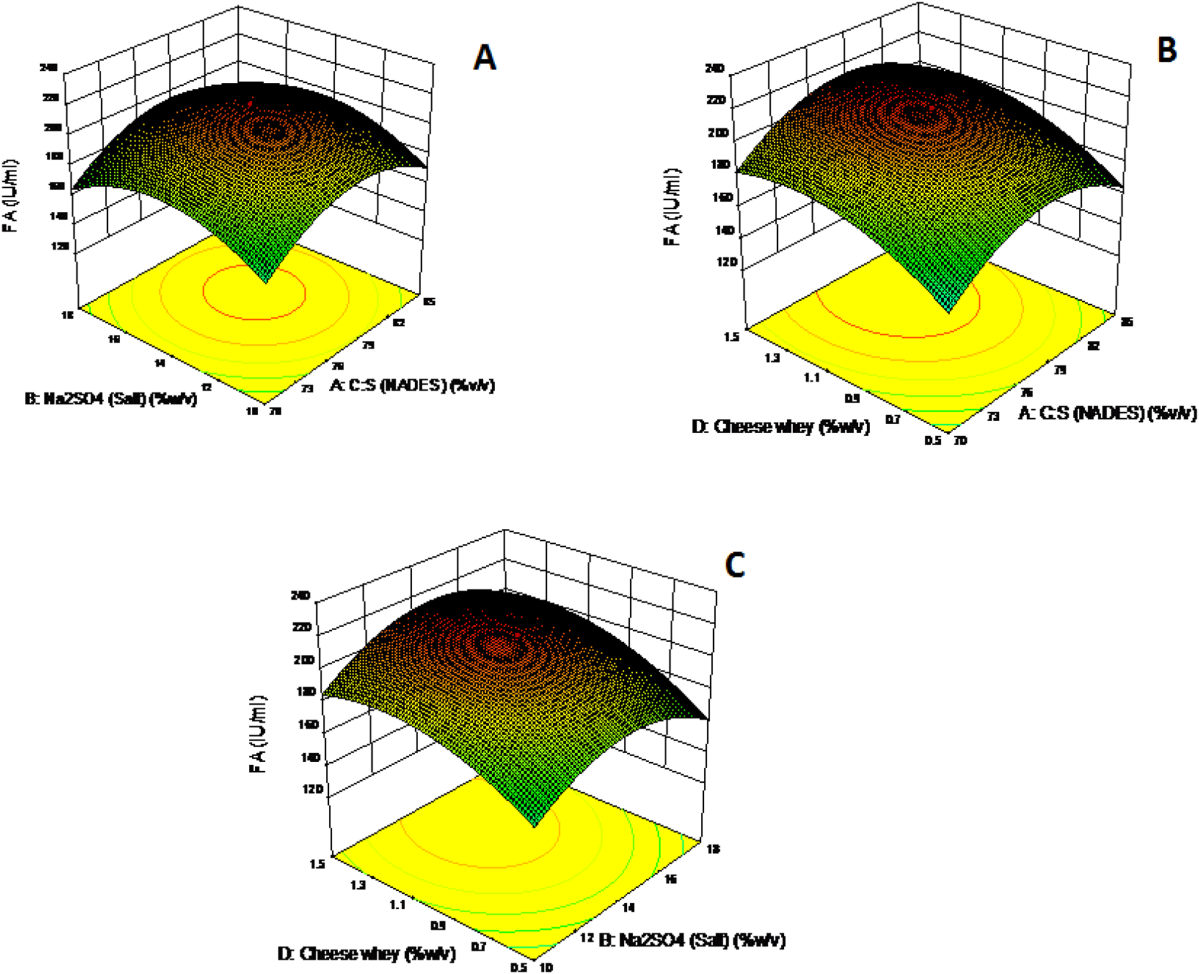
(**A**) Response surface plot showing the effect of salt concentration and nitrogen source (cheese whey) on fibrinolytic enzyme production. (**B**) Response surface plot showing the effect of nitrogen source (cheese whey) and DES concentration on fibrinolytic enzyme production. (**C**) Response surface plot showing the effect of nitrogen source (cheese whey) and salt concentration on fibrinolytic enzyme production.

The species under investigation for the integrated production of fibrinolytic protease was *Bacillus cereus.* The extractive fermentation model was constructed and analyzed based on the aqueous two-phase system established with NADES. All the independent variables that were chosen to be optimized show high influence on the recovery of fibrinolytic protease. The enzyme activity in the top phase ranges between 121.3 and 218 IU/ml. From the (Fig. 5), it was noticed that the NADES concentration and the concentration of nitrogen in growth medium are the major influential factors. The optimum enzyme activity of about 217 IU/ml was achieved with the biphasic system formed by M:S (77.5% w/v) and Na_2_SO_4_ (14% w/v) and at nitrogen source value of 1% (w/v). The acquired values were evaluated using Central Composite Design (*p* > 0.0001, f = 250.45, R-squared value = 0.93 Lack of fit = 2,401.38). In all the aqueous two-phase system formed with NADES, the cell debris settled in the raffinate phase with other undesirables while the protein of interest was found to be enriched in the top phase. The earlier investigation reported an increased yield of 1,223 IU/ml^3^, but the process involved the usage of polyethene glycol which poses trivial problems during purification of the enzyme. The extraction of alkaline protease was reported in the literature^31^ provides a high yield of 2,856 IU/ml in both the phases which makes the process non-selective and non-viable. The present investigation uses NADES as solvent counterpart assisting the formation of aqueous two-phase which guarantees effluent free operation. Additionally, industrial byproducts like cheese whey have been used in the production of the therapeutic enzyme which has satisfied the concept of the circular economy. Thus, the present investigation ensures sustainability in the production of fibrinolytic protease.

### Quantification of fibrinolytic protease by gel filtration chromatography

**Figure 6:**
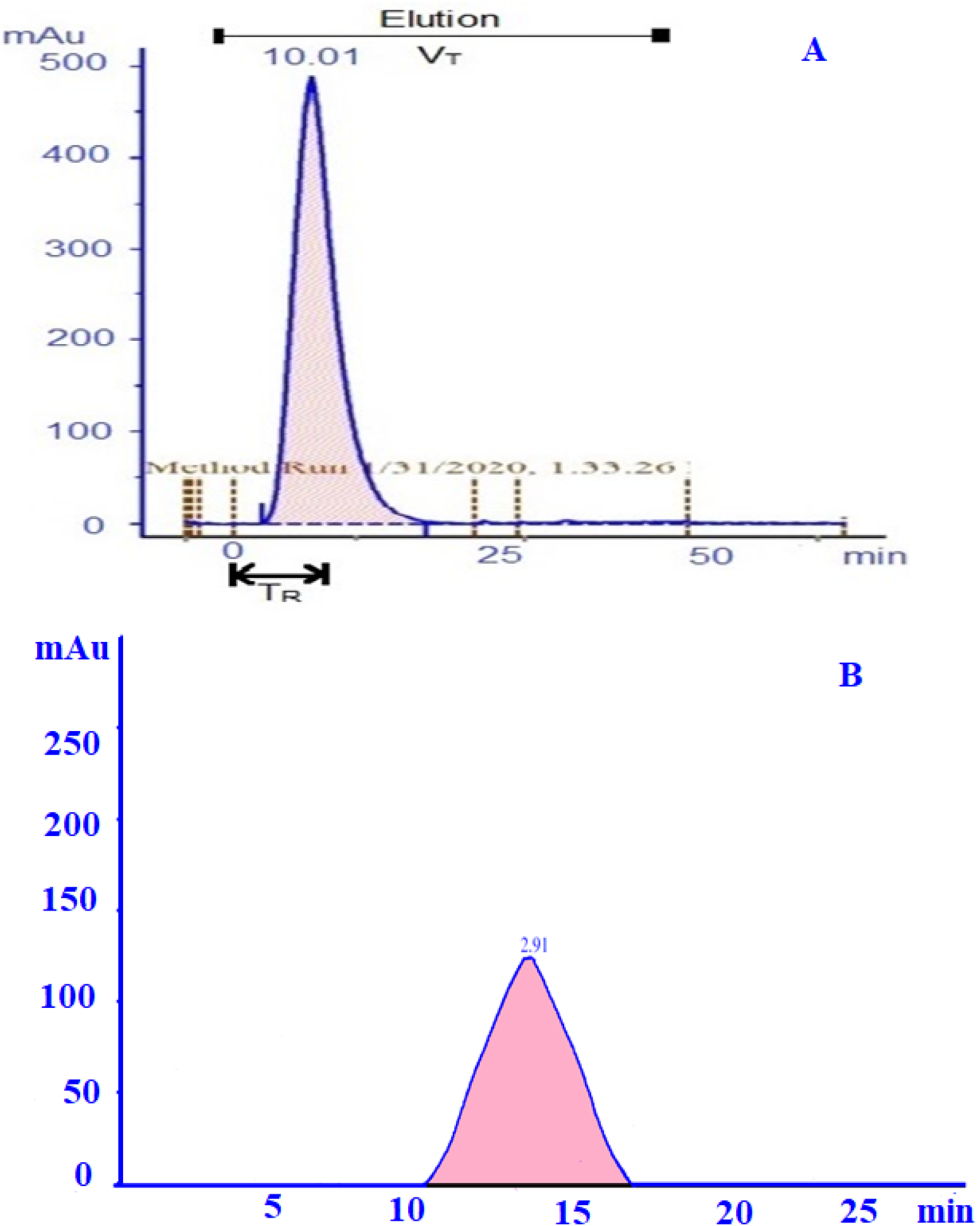
(**A**) Chromatogram of gel filtration chromatography performed with Sephadex G-15 column at the flow rate of 1 ml/min which displayed a residence time of 10 min (approximately). Tris HCl buffer at 20 mM was used as the elution buffer at a pH of 7.5. T_R_ indicates the retention time and V_T_ indicates the elution volume. (**B**) Chromatogram of anion exchange chromatography performed with sepharose column at the 1 ml/min. 20 mM Tris HCl buffer used as binding buffer and 1 M NaCl used as elution buffer.

The sample collected from extractive fermentation was quantified and purified with a size exclusion column (Sephadex G-15, GE AKTA prime plus) The process parameters were optimized for high yield with enhanced purification. The eluted fractions exhibiting a single peak is observed to be rich in an active enzyme (protease) and was observed to occur at a retention time of 10 min. The flow rate maintained was 1.0 ml/min throughout the process to reduce discrepancy during purification^32^. The total retention volume was calculated to be 24.03 ml (Fig. 6A). The number of theoretical plates which implies separation efficiency was observed to be 2.7 NTU. The active fractions collected by fraction collector exhibits the high fibrinolytic activity of 190 U/ml.

### Ultra-purification using anion-exchange chromatography

After pre-equilibration of the anion exchange column (DEAE-sepharose, GE AKTA prime plus), the sample was injected at a constant flow rate of 1.5 ml/min. This ionic environment DEAE is due to the fine tuning of the column to the pH of 8.8 buffer reported by^33^. The strong interaction developed facilitates selective enzyme binding. On subsequent equilibration using the binding buffer, unwanted proteins that do not bind to the matrix gets washed away. Further, the passage of elution buffer forms an increased concentration gradient of salt and elutes the bound peptide from DEAE. It is evident from the chromatogram that equilibration of the column before the injection of the sample was effective. After injection of the sample, a tiny peak is observed before 1/3 of column volume indicating the impurity removal. Following that, a peak with active fractions was observed to start with a retention time of 8 min. Since the whole process is run in a similar flow rate there is no difference in the retention volume and thus the yield is calculated. The yield of fibrinolytic protease with minimal error function value of 95% void volume is obtained. The retention time (t) is 11 min and maximum retention occurs at (t_max_) of 10.2 min while σ_tmax_ of 1.5 ml yielding 88% of active fractions recovered (Fig. 6B).

### Native PAGE of fibrinolytic fractions

**Figure 7:**
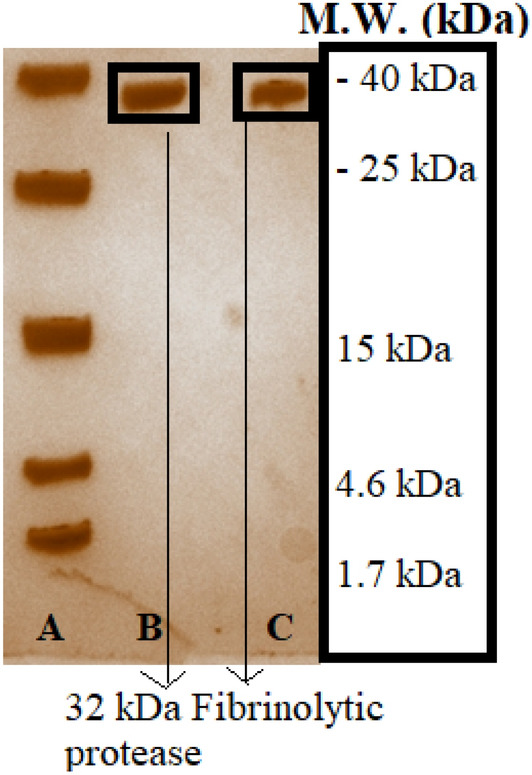
Native gel electrophoresis of fibrinolytic protease fractions collected from (**B**) optimised extractive fermentation and (**C**) ultrapure fractions fro anion exchange chromatography being loaded along with (**A**) low weight molecular weight marker.

Figure 7 denotes the resulting native gel with protein bands for respective fractions of the product occurring from extractive fermentation and anion exchange chromatography respectively. A single protein band is observed to occur for both fractions and the latter one (C) seems to carry a sharp band due to ultra-purity of the fraction. This denotes the occurrence of pure fibrinolytic protease by extractive fermentation whose purity is further enhanced by chromatography. Both these bands tend to occur identical to the molecular weight marker band of 32 kDa size which represents the molecules obtained are in the molecular weight range of 20–25 kDa matching with the previously identified size of fibrinolytic protease.

### Enzyme analysis

#### Effect of pH and temperature on enzyme activity and enzyme stability

**Figure 8:**
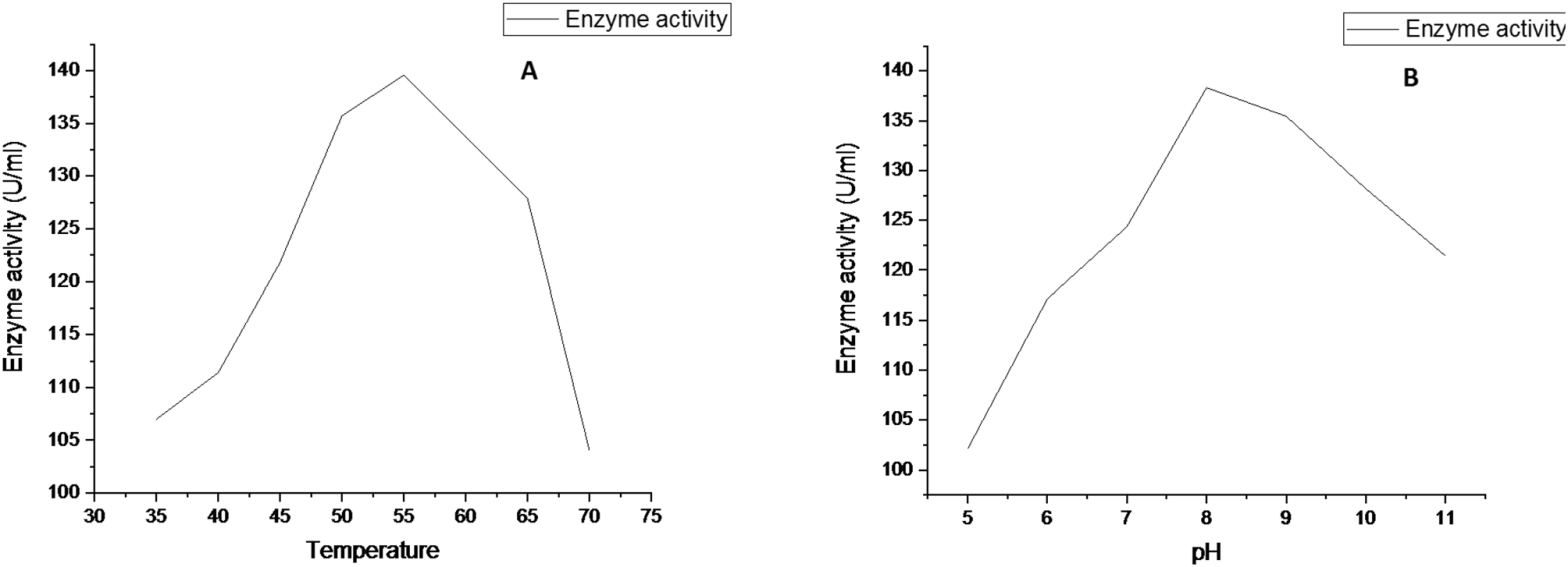
The graph (**A**) shows the activity of enzyme was high and optimitized at pH 8. The graph (**B**) shows the activity of fibrinolytic enzyme is high and optimized at temperature 55 °C. The enzymes were maintained at constant agitation of 150 RPM for 24 h for both the experiments.

The purified enzyme activity was evaluated with the buffers of different. It shows that the enzyme becomes active over a board range of pH 7.5–12. The maximum enzyme activity was observed at pH 8 (Fig. 8A). The temperature influence on the purified enzyme was observed between 30 and 55 °C, the maximum enzyme activity was observed at 50 °C (Fig. 8B). At a higher temperature of 80 °C, the reduced enzyme activity was noted and the results occur in corrugation with earlier investigation^34^.

#### Effect of substrate concentration on enzyme activity

**Figure 9:**
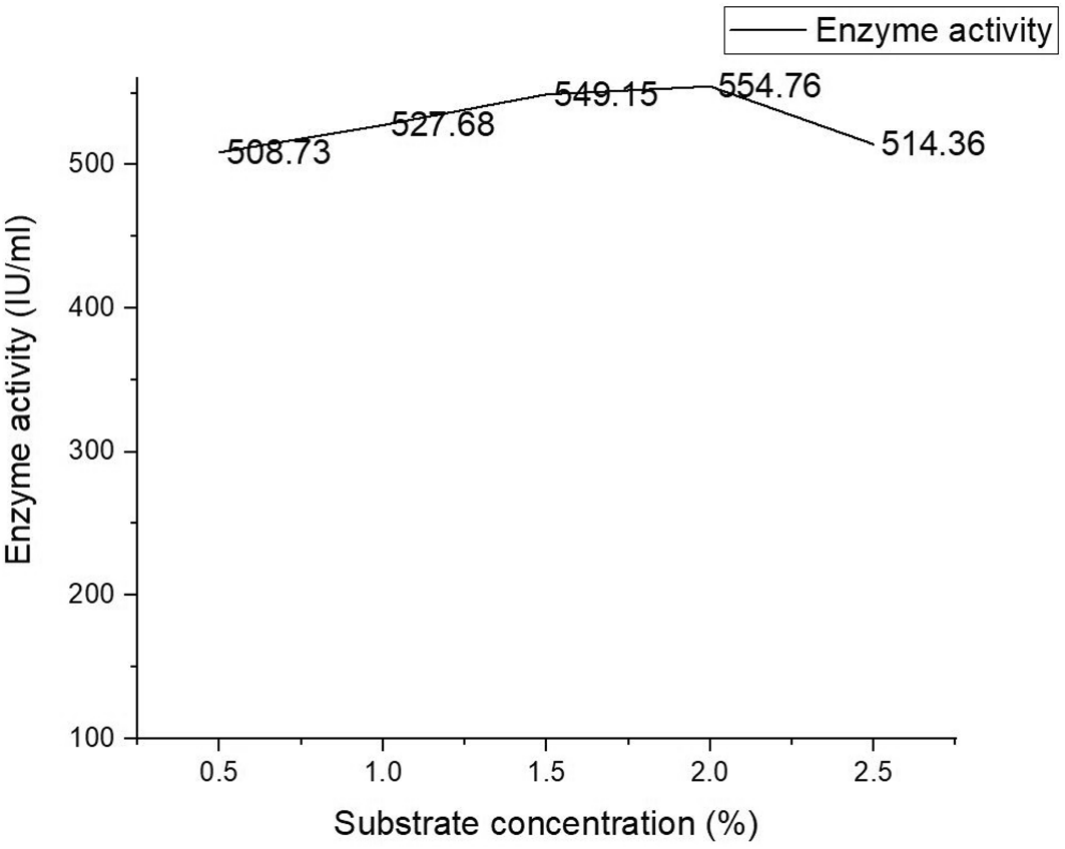
The graph shows the enzyme activity at different substrate concentration. The maximum enzyme production was obtained at 2% substrate concentration.

From Fig. 9 it could be observed that the optimal substrate concentration for attaining maximum enzyme activity of the ultrapure protease fraction was around 2% (w/v). This concentration very low compared to the previous investigations because the purity fold of the enzyme has increased which has enhanced its specificity.

### Fibrin plate assay

**Figure 10:**
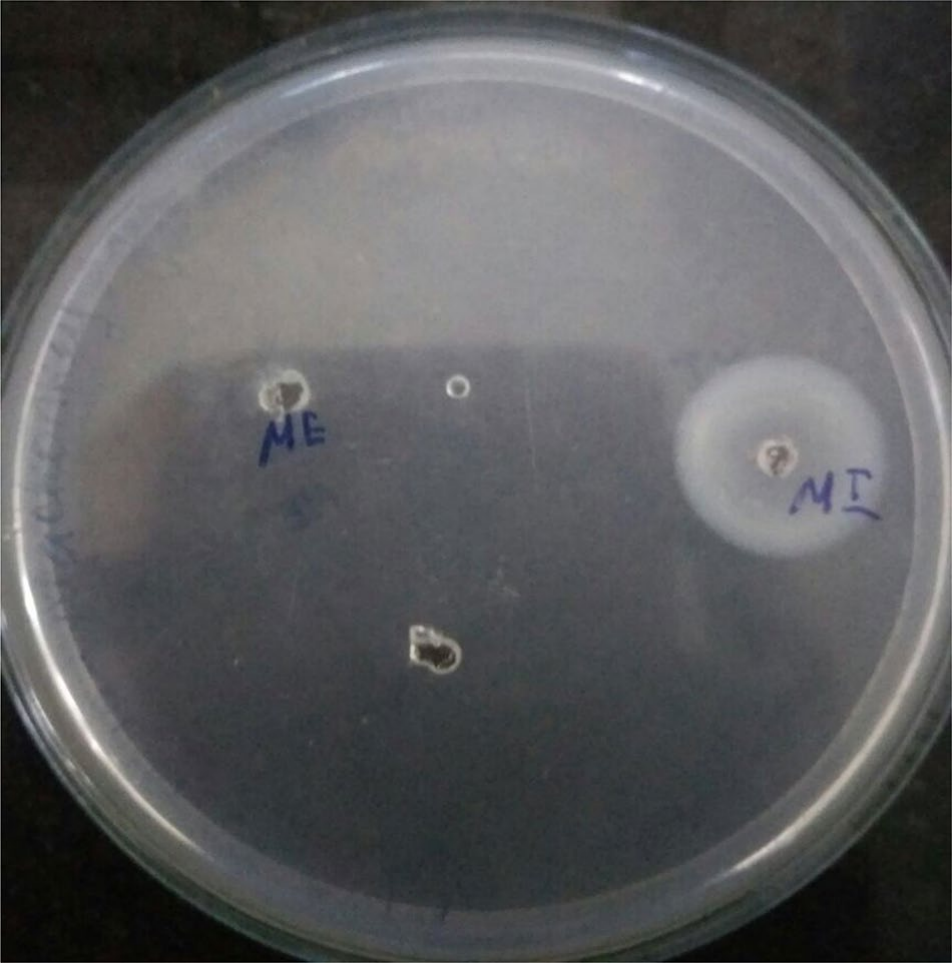
Picture shows the clear zone of hydrolysis due to the fibrinolytic activity of enzyme produced by *Bacillus cereus* ATCC 14,579 using fibrinolytic enzyme volume of 10 µl. The plate was incubated at 37 °C for 18 h.

Both plasminogen rich fibrin plate and plasminogen free fibrin plate was used with minor modifications for the determination of fibrinolytic activity. Plasminogen rich fibrin plate was made up of fibrinogen solution (5 ml of 0.5% human fibrinogen in 20 mmol Tris-HCl buffer at pH 7.5), 20 U of thrombin solution and 5 ml of 1% agarose in Petri dishes (5 cm in diameter). In the case of plasminogen free fibrin plate method, the plate is heated to 80 °C for 30 min to destroy other fibrinolytic factors^35^. For the observation of the fibrinolytic activity 10 μl of the enzyme is carefully dropped in the fibrin plate. The plate is kept at 37 °C for 18 h and the activity is observed (Fig. 10).

## Materials and methods

### Chemical and reagents

Menthol (M) (2216-51-5) and bovine serum albumin (9048-46-8) and other assay chemicals were purchased from Sigma-Aldrich USA with 97% purity. Glucose (G) (50-99-7), fructose (F) (57-48-7), xylose (X) (58-86-6), maltose (M) (69-79-4), lactose (L) (63-42-3) and sucrose (S) (57-50-1), Na_2_SO_4_ (7757-82-6), K_2_HPO_4_ (7758-11-4), Na_2_CO_3_ (497-19-8)and growth medium (Luria–Bertani broth media (M1245)) were obtained from Himedia, India with a purity standard greater than 95%. All the chemicals used and their purification method is detailed in Table 3.

**Table 3:** Source and purity various chemicals used for DES synthesis.

Chemical name	CAS reg. no.	Supplier	Mass % purities stated by suppliers	Purification method
Menthol	2216-51-5	Sigma-aldrich	0.99 w/w	Overnight storage in dessicator
Sodium sulphate	54664-61-8	Himedia	0.97 w/w	Overnight storage in dessicator
Potassium phosphate	7758-11-4	Himedia	0.97 w/w	Overnight storage in dessicator
Sodium carbonate	497-19-8	Himedia	0.97 w/w	Overnight storage in dessicator
Glucose	50-99-7	Sigma-aldrich	0.99 w/w	None
Fructose	58-49-7	Sigma-aldrich	0.99 w/w	None
Sucrose	57-50-1	Merck	> 98 w/w	None
Xylose	609-06-3	Merck	> 98 w/w	None
Maltose	69-79-4	Merck	> 98 w/w	None
Lactose	63-42-3	Merck	> 98 w/w	None

### Sample collection

Lyophilized culture of *Bacillus cereus* (strain number-14579) we purchased from MTCC, Chandigarh. These were plated and incubated for 24 h at 37 °C for the screening of protease activity^36^. These protease producing colonies were enriched in casein plates until a single colony of the bacteria was obtained. Complex media sources of fibrinolytic protease production were selected from different localities in Thanjavur. Cane molasses were collected from sugarcane industry at Thanjavur, Rice water was collected from rice steaming unit Tiruchirappalli, cheese whey was collected from a cottage industry, Tiruchirappalli. Collected sources were pre-treated with 20 Mm Tris-HCl buffer and preserved in the sterile container for further use.

### Screening and complex media preparation

A diverse variety of complex medium sources such as rice water, molasses, cheese whey and rotten egg were chosen as substrates for bacteria. Medium was prepared by adding, cheese whey^37^ (1% v/v), glucose (0.5% w/v); K_2_HPO_4_ (0.4% w/v); Na_2_HPO_4_ (0.1% w/v); CaCl_2_ (0.01% w/v); Na_2_CO_3_ (0.6% w/v); MgSO_4_·2H_2_O (0.01% w/v^38^, supplements in 100 ml distilled water. The prepared media was sterilized at 15 psi and 121 °C. The inoculum of 1 ml volume was transferred from seed culture and was incubated for 24 h at 150 rpm in refrigerated shaker incubator (REMI, C plus).

### Synthesis and thermophysical characterization of NADES

NADESs were synthesized by mixing individual hydrogen bond acceptor (HBA) corresponding with hydrogen bond donor (HBD) in the appropriate molar ratio. The mixture was heated above 80 °C to form the eutectic solvents. Thermophysical parameter such as density, conductivity, viscosity and refractive index of the NADESs was measured in terms of varying temperature (273–323 K). The density of the NADESs was estimated using density meter (DDM 2910) by Rudolf, USA. The refractive index was measured for the NADESs with a handheld refractometer Atago (MASTER-PM). Brookfield LV II + Digital viscometer was used to measure the viscosity of all the NADES in the temperature range of *298–*323 K (± 0.5 K). All these experiments were performed thrice to determine the mean along with the standard deviation^29,39^.

All NADES formed were examined for the presence of their functional groups of corresponding HBA and HBD through H^1^ NMR analysis. The NMR analysis is performed by dissolving 8 mg equivalent of the sample in 500 µl of D_2_O in the quartz tubes provided and ultra sonicated for even dissolution. The measurements were done with 300 MHz BRUKER AVANCE II (Bruker Biospin, Switzerland) spectrometer equipped with 5 mm BBO probe. The resonance was recorded at 298.15 K and processed using pulse sequence library of Topspin 3.2. (Bruker biospin, Switzerland).

The binodal curve of NADES was observed with Na_2_SO_4_ as the salting-out agent. Initially, 5 ml volume of 80% (v/v) of NADES is taken in a separation funnel to which Na_2_SO_4_ solution 20% (w/v) was added dropwise until the solution becomes turbid denoting the formation of first cloud point. The system is left undisturbed for a few minutes until two significant phases were observed. Aliquots of samples from individual phases were aspirated and the mass of individual components (NADES and Na_2_SO_4_) was recorded with Shimadzu BL-220H. Successive cloud points were formed by diluting the former cloud point with water followed by the addition of salt solution to form turbidity. The observations were plotted with the mass ratio of NADES in X-axis and Na_2_SO_4_ in Y-axis. This plot denotes the bimodal curve of the individual NADES.

### Extractive fermentation of protease

The inoculated broth pre-incubated at 37 °C, pH 7 for 21 h batch time was used for extractive fermentation. Sterile NADES (90% v/v) was added to the culture and the addition of salt (20% w/v) at a particular composition promotes the formation of cloud point. The added components were mixed in a magnetic stirrer for equilibration and left for phase separation at room temperature for 2 h. After the formation of clear boundaries, the top phase was removed and evaluated for recovery of protease.

Selection of solvents promoting better phase formation was done with six NADES and various salts of choice (Na_2_SO_4_, Na_2_CO_3_ and K_2_HPO_4_). The yield of the product corresponding to its high phase ratio depends on a combination of the salt and individual NADES. Phase ratio of ATPS for each combination of NADES and salt was estimated by the following equation.

4 $$ P = \frac{{V_{t} }}{{V_{b} }} $$

where V_t_ indicates the volume of the top phase and V_b_ indicates the volume of the bottom phase of the system. Each value was measured in triplicates and the mean along with standard deviation was determined for individual samples.

The partition coefficient of six NADES was screened for evaluating the selectivity of NADES used. Higher partition coefficient denotes that the NADES under investigation is more selective towards the extraction of the target enzyme to the top phase. The top phase and bottom phase of the mixture were collected separately and the amount of enzyme recovered in each phase was measured by casinolytic assay. The partition coefficient was calculated using the following equation.

5 $$ K = \frac{{C_{t} }}{{C_{b} }} $$

where K is the partition coefficient, C_t_ and C_b_ are the enzyme concentration corresponding to top and bottom phase respectively.

### Optimization of growth media and extraction components for extractive fermentation

The influence of essential variables over effective phase separation of fibrinolytic protease was optimized using response surface methodology. The statistical evaluation using central composite design for the different variable under investigation was achieved with Design Expert (v10.1 Stat-ease, Minneapolis, USA). The variables chosen to be optimized were NADES concentration (70–85% w/v) (A), Salt concentration (10–18% w/v) (B) and source nitrogen concentration (0.5–2.5% w/w)^40^ (D). The total of 32 runs that including 6 center points, 2 axial and factorial points was carried out. The yield of the fibrinolytic enzyme in the top phase was chosen as the response variable. For conducting individual experiments the media at defined composition was taken in a 250 ml Erlenmeyer flask (pH 8.0), inoculated with seed culture of 1% v/v and incubated at 50 °C; 150 rpm for a batch time of 24 h. Further, NADES and salt at defined concentration were added until cloud point formation and the system was kept stationary until the formation of a distinct phase. Finally, the amount of active enzyme recovered in each phase was determined by evaluating the fibrinolytic activity.

### Back-extraction

Considering the factor of green separation the solvent used should be greener and recyclable. DES used in the process of extraction was recovered back by treating them with optimized concentrations of salt under certain concentrations. The mechanism of reverse extraction is a simple process that involves the disruption of vanderwaal’s interaction between the target peptide and the DES at optimum conditions. Previous investigations revealed that the effect of product recovery increases minimally with the agitation speed, thus the agitation was set to 100 rpm throughout the process. Different salts (NaCl and KCl) at various concentrations (10–15%) at a temperature of 5–15º C and agitation for about 1–4 h maintained during the process.

### Preparative size exclusion chromatography for isolation of fibrinolytic protease

Size exclusion chromatography was performed to isolate proteolytic enzyme with fibrin degrading activity. The Crude extract containing the enzyme was isolated from the top phase of the aqueous biphasic system and loaded onto the sample port. The column (Sephadex G-15, GE AKTA prime plus) of 5 ml bed volume was used for the process. Before loading the sample, the column was equilibrated with 20 mM phosphate buffer (pH 7) at a constant flow rate of 1 ml/min maintained throughout the separation process^41^. The sample was injected into the sample port which penetrates and runs through the column along with the binding buffer. Elution of bound metabolite was carried out by passing the elution buffer (20 mM phosphate buffer, pH 7.0, 1 M NaCl) as a linear gradient with the binding buffer. The non-target large protein molecule moves rapidly through the column while the target protein takes time for elution. Individual fractions corresponding to the peaks observed in the chromatogram was collected in fraction collector and analyzed for fibrinolytic activity. The fraction exhibiting maximum fibrinolytic activity was regarded to be the isolate with the product of interest.

The separation efficiency of the process for fibrinolytic protease purification was evaluated by calculating the number of theoretical plates formed between the mobile phase and the Sephadex matrix. The number of theoretical plates formed is measured by the following equation.

6 $$ n = 16\left( {\frac{{t_{r} }}{{w_{b} }}} \right)^{2} $$

n—Number of theoretical plates; t_r_—retention time; w_b_—length of baseline for the elution peak.

The retention volume was calculated as the volume of buffer required to elute the bounded enzyme. The affinity between the sample and the stationary phase was determined based on the retention volume of the pure fraction.

7 $$ V_{R} = t_{R} \times W_{b} $$

V_R_—Retention volume; t_R_—Retention time; W_b_—the width of the base of the peak.

### Ultra purification using anion-exchange chromatography

The active fractions collected from size exclusion chromatography were ultra-purified using an anion exchange column (DEAE-sepharose, GE AKTA prime plus) with 5 ml bed volume. The column was pre-equilibrated with equilibrating buffer (20 mM Tris-HCl, pH 8.0) and the flow rate was maintained at 1 ml/min. Equilibration of the column was done until the detector shows zero baseline. The sample was loaded into the sample port enabled with 500 µl sample loop. Another cationic molecule that was similar in charge with molecules of matrix doesn’t bind with the stationary phase and flow rapidly through the column. The molecules bound to the matrix were eluted using elution buffer (20 mM Tris-HCl, pH 8.0, 1 M NaCl) forming a linear gradient with the equilibration buffer^42^. While the undesirables were eluted with the equilibration step, the target molecule with the isoelectric point in congruence with the pH of the binding buffer which was bound to the active matrix was eluted along with the passage elution buffer. The yield of ultrapure enzyme thus occurring can be calculated with the following equation.

8 $$ Yield\; of\; enzyme = \frac{1}{2}\left[ {1 + {\text{error function}}\left( {\frac{{t - t_{max} }}{{\sqrt 2 \sigma_{tmax} }}} \right)} \right] $$

The active fraction showing maximum absorbance in the UV detector is collected.

### Native PAGE electrophoresis

Native gel electrophoresis was performed for the active fractions obtained from extractive fermentation and anion exchange chromatography. Mini protean-II cell (Biorad, F-94203) was used for performing the electrophoresis with stacking (4% w/v) and separating (12% w/v) gels prepared with acrylamide/bis acrylamide mixture respectively and laid one over the other. Spectra Low range molecular weight (Thermofischer scientific) was used as molecular weight marker. Post loading of samples, the electrophoresis was run at 100 V for 1 h and the resultant gel is stained using silver nitrate solution (12 mM).

### Enzyme analysis

#### Effect of pH and temperature on enzyme activity

The enzyme activity at different pH was measured by suspending the enzyme in various buffers ranging from 5.0 to 11.0. The buffers used were sodium acetate (pH 5.0), phosphate buffer (pH 7.0) and Tris-HCl (pH 8.0). All tubes preloaded with 2 ml of these pH buffers were incubated along with ultrapure enzyme fraction (0.1 ml) at room temperature for 2 h. The effect of temperature on enzyme sample was determined in the range of 30–50 °C^43^ by suspending 0.1 ml of ultrapure enzyme fraction in 2 ml of 0.1 M Tris-HCl buffer (pH 9.0) and incubating at different temperature for 2 h. Post incubation, aliquots of enzyme suspension (400 µl) from all tubes were mixed with 1 ml of 1% (w/v) of casein solution (phosphate buffer, pH 7.5) and incubated at room temperature for 30 min. Following incubation, 500 µl of 10% (w/v) TCA was added and the tubes were centrifuged at 8,000 rpm for 10 min. 2 ml of the supernatant was mixed with 5 ml of sodium carbonate (0.5 M) and 1 ml of (0.2 M) folins reagent and incubated at room temperature for 30 min. The resultant mixture was measured at 660 nm. A standard plot was drawn between the known concentration of tyrosine against its absorbance and the concentration of tyrosine released from casein using the unknown concentration of enzyme was determined. The amount of enzyme present in the fraction was calculated by the following equation with the amount of tyrosine released.

9 $$ \frac{Units}{{ml \;enzyme}} = \frac{{\left( {\mu mol \;of\; tyrosine\; equivalents \;released} \right)* \left( {11} \right)}}{{\left( 1 \right)*\left( {10} \right)*\left( 2 \right)}} $$

#### Effect of substrate concentration on enzyme activity

According to the Michaelis–Menten equation, increasing the amount of substrate increases the rate of enzyme activity until it reaches a maximum value (V_max_). 2 ml of casein was mixed with 0.1 ml of enzyme at various concentrations (0.5%, 1%, 1.5%, 2%, 2.5%) and incubated at 37 ºC for 10 min. Post incubation, the reaction was arrested by the addition of 3 ml of 10% TCA and mixture was centrifuged. To 2 ml of supernatant, 5 ml of sodium carbonate (0.5 M) and 1 ml of (0.2 M) folins reagent was added and incubated for 30 min at room temperature. The absorbance of the resulting dark blue solution was recorded at 660 nm. The reaction velocity (V) was defined as the rate of increase in absorbance at 660 nm. By plotting the graph of the inverse of substrate concentration to the inverse of V the value of K_m_ and V_max_ could be estimated.

10 $$ V_{0} = \frac{{V_{max} \left[ S \right]}}{{K_{m} + \left[ S \right]}} $$

## Conclusion

The present investigation reports for the first time the production and purification of the fibrinolytic protease from *B. cereus* have established by extractive fermentation. Fibrinolytic enzyme was obtained at the top phase of ATPS formed with M:S (77.5% w/v) and Na_2_SO_4_ (14% w/v). The optimum enzyme activity was found to be 217 IU/ml by response surface methodology. The maximum yield achieved by the extractive fermentation was 93.6% (Table 4). The quantification of the fibrinolytic enzyme was accomplished with gel filtration chromatography and purification of the enzyme was accomplished with anion-exchange chromatography. Further, the obtained pure product was subjected to enzyme kinetics. The optimal activity of the enzyme was obtained at pH 8 (50 °C). The coupling of the fermentation and downstream process helps to increase the purity of the product in single unit operation. The present investigation uses industrial byproducts like cheese whey for production of this therapeutic enzyme and its purification was accomplished with naturally derived NADES which has been recycled reducing the effluent during downstream processing. Thus, this study has aided in the sustainable production of fibrinolytic protease satisfying theory of circular economy.

**Table 4:** Increased purity fold and decreased total protein recovery by different downstream steps for fibrinolytic proteases (n = 3, mean values represented along with standard deviation) (*p* < 0.05).

Downstream process	Total protein (mg/ml)	Enzyme activity (U/ml)	Specific activity (U/mg)	Yield (%)	Purity fold
Crude	417.54	23,131.85	55.4	100	1
Extractive fermentation	98.5	21,670.0	220	93.6	3.97
Gel filtration chromatography	10.5	7,917.0	754	34.2	13.6
Anion exchange chromatography	2.21	2,607.8	1,180	11.2	21.2

## Supplementary information

Supplementary Information 1.
