# Supplementary material for: One-pot simultaneous production and sustainable purification of fibrinolytic protease from Bacillus cereus using natural deep eutectic solvents
Source: Sci Rep. 2020 Aug 7;10:13356. doi: 10.1038/s41598-020-70414-2 (PMC7414877; doi:10.1038/s41598-020-70414-2)
Supplement: Supplementary file 1 — Supplementary Information 1. [file 41598_2020_70414_MOESM1_ESM.docx]

**Supplementary information**

**One-pot simultaneous production and sustainable purification of fibrinolytic protease from *Bacillus cereus* using natural deep eutectic solvents**

Senthilkumar Rathnasamy ^1C*^, Aadhavan Durai ^1A^, Vigneshkumar A A. ^1A^, Purushothaman C. ^1B^, Devi Sri Rajendran^1A^, Chandramouliswaran K ^1B^.

^1^Bioseparations Laboratory,

School of Chemical and Biotechnology,

SASTRA Deemed to be University, Thanjavur, Tamil Nadu – 613401

^A^Experimental investigation

^B^Writing and analysis

^C^Conceptualization, methodology, funding acquisition, and project  supervision

^*^Corresponding Author

Senthil Kumar Rathnasamy,

Assistant Professor, Bioseparations Laboratory,

SASTRA Deemed to be University, Thanjavur, Tamil Nadu - 613401

Email address: [senthilrathna@sastra.ac.in](mailto:senthilrathna@sastra.ac.in)

Figure S1: H^1^ NMR spectrum of NADES prepared by mixing Menthol with lactose in molar ratio of 1:1

Figure S2: H^1^ NMR spectrum of NADES prepared by mixing Menthol with Glucose in molar ratio of 1:1

Figure S3: H^1^ NMR spectrum of NADES prepared by mixing menthol with fructose in molar ratio of 1:1

Figure S4: H^1^ NMR spectrum of NADES prepared by mixing menthol with xylose in molar ratio of 1:1

Figure S5: H^1^ NMR spectrum of NADES prepared by mixing Menthol with maltose in molar ratio of 1:1

Figure S6: H^1^ NMR spectrum of NADES prepared by mixing Menthol with Sucrose in molar ratio of 1:1
